# A Thermostable, Modified Cathelicidin-Derived Peptide With Enhanced Membrane-Active Activity Against *Salmonella enterica* serovar Typhimurium

**DOI:** 10.3389/fmicb.2020.592220

**Published:** 2021-01-13

**Authors:** Natthaporn Klubthawee, Ratchaneewan Aunpad

**Affiliations:** Graduate Program in Biomedical Sciences, Faculty of Allied Health Sciences, Thammasat University, Pathum Thani, Thailand

**Keywords:** thermostable peptide, drug-resistant *Salmonella*, membrane-active mechanism, induction of resistance, food preservative

## Abstract

Foodborne illness caused by consumption of food contaminated with *Salmonella* is one of the most common causes of diarrheal disease and affects millions of people worldwide. The rising emergence and spread of antimicrobial resistance, especially in some serotypes of *Salmonella*, has raised a great awareness of public health issues worldwide. To ensure safety of the food processing chain, the development of new food preservatives must be expedited. Recently, thermal- and pH-stable antimicrobial peptides have received much attention for use in food production, and represent safe alternatives to chemical preservatives. A 12-mer cathelicidin-derived, α-helical cationic peptide, P7, displayed rapid killing activity, against strains of drug-resistant foodborne *Salmonella enterica* serovar Typhimurium and its monophasic variant (*S. enterica* serovar 4,5,12:i:-) and had minimal toxicity against mouse fibroblast cells. P7 tended to form helical structure in the membrane-mimic environments as evaluated by circular dichroism (CD) spectroscopy. The action mode of P7 at the membrane-level was affirmed by the results of flow cytometry, and confocal, scanning and transmission electron microscopy. P7 killed bacteria through binding to bacterial membranes, penetration and the subsequent accumulation in *S. enterica* serovar Typhimurium cytoplasm. This induced membrane depolarization, permeabilization, and sequential leakage of intracellular substances and cell death. Except for sensitivity to proteolytic digestive enzymes, P7 maintained its inhibitory activity against *S. enterica* serovar Typhimurium in the presence of different conditions [various salts, extreme pHs and heat (even at 100°C)]. Moreover, the peptide is unlikely to induce bacterial resistance *in vitro*. Taken together, this study demonstrated that the membrane-permeabilizing P7 peptide has much potential as a new antimicrobial agent for use in food processing and preservation.

## Introduction

*Salmonella* are gram-negative bacteria and common causes of diarrheal diseases affecting millions of people all over the world, and notably some resistant serotypes have emerged. *Salmonella enterica* serovar Typhimurium (*S. enterica* serovar Typhimurium) is the most frequently reported serovar causing human salmonellosis worldwide (Bugarel et al., 2012; Gunell et al., 2014). In recent decades, a monophasic variant of *S. enterica* serovar Typhimurium (named *S. enterica* serovar 4,5,12:i:-) that is deficient in the expression of the phase-2 flagellar antigen has emerged. This variant is a growing public health problem because of its drug resistance, pathogenicity, existence among food and veterinary chains, and capability to spread extensively (Arai et al., 2018; Mastrorilli et al., 2018). Consumption of contaminated foodstuffs, especially eggs, meat, poultry, and milk, is the major transmission route of human *Salmonella* infections. Besides food hygiene practices, such as “cook thoroughly,” recommended by the WHO as a basic preventive strategy (World Health Organization [WHO], 2018), preservative agents are important in keeping food safe and unspoiled by microorganisms.

Antimicrobial peptides (AMPs) have recently garnered increased interest as promising food preservatives to improve on the limitations of common chemical preservatives which may have adverse effects on human health, and increase the frequency of multidrug-resistant pathogens (Rai et al., 2016; Thery et al., 2019). The European Food Safety Authority (EFSA) confirmed that nisin, an authorized food preservative peptide, can be used safely as a food additive in the proposed expansion use in unripened cheese and heat processed meat products (EFSA Panel on Food Additives et al., 2017). Nisin has very potent activity against Gram-positive bacteria by binding to lipid II and forming membrane pores, however, its activity against Gram-negative bacteria is much lower due to the impermeable outer membrane and the inner membrane localization of lipid II (Li et al., 2018). Moreover, spontaneously acquired nisin-resistant isolates of foodborne pathogens were recently found and characterized (Bucur et al., 2018; Szendy et al., 2019). Several derivatives of cationic α-helical peptides were designed from the cathelicidin family, for example porcine PMAP (Lv et al., 2014) and human LL-37 (Chen et al., 2019). They show promise to be developed as new antimicrobial agents against bacterial pathogens. This has led to a search for new α-helical cationic AMPs as alternative preservatives which are safe, exhibit broad spectrum, and are unlikely to induce antimicrobial resistance.

Recently, a 12-mer α-helical, cationic AMP (‘P7’) was designed based on the cathelicidin family using a template-modified strategy, and subsequently modified by truncation and amino acid substitution (Klubthawee et al., 2020). P7 displays potent antibacterial activity against both Gram-negative and Gram-positive bacterial pathogens, notably including *S. enterica* serovar Typhimurium. Of a series of peptides, P7 exhibited the highest therapeutic index (TI) among a series of peptides in terms of its activity toward bacterial cells compared with human red blood cells (hRBCs). Furthermore, P7 shows no obvious hemolytic activity (less than 5% hemolysis) even at the highest concentration tested (250 μg/ml) (Klubthawee et al., 2020). In order to assess its potential use and effectiveness, as well as its mechanism of action, the antimicrobial activity of P7 against 24 drug-resistant foodborne and 11 drug-resistant clinical *S. enterica* serovar Typhimurium or 4,5,12:i:- isolates, were evaluated and compared to that of nisin. Its mechanism of antibacterial action at membrane-level was studied alongside its stability under different conditions such as various salts and enzymes, and extremes of pH and heat. In addition, the induction of bacterial resistance to the P7 peptide was explored. We highlighted the potential use of P7 as an alternative agent for food preservation, prolonging shelf-life, and the wider food processing industry.

## Materials and Methods

### Peptide Synthesis

All peptide derivatives, including amidated P7 (KIAKRIWKILRR-NH_2_) (Klubthawee et al., 2020) and tetramethylrhodamine (TAMRA)-labeled P7, were synthesized by China Peptides Co., Ltd (Shanghai, China) with solid-phase methods using 9-fluorenylmethoxycarbonyl (Fmoc) chemistry. TAMRA-labeled P7 was prepared using dehydration condensation by linking TAMRA with the N-terminus of P7 via an amide bond. TAMRA-labeled P7 was chosen to determine the membrane-penetrating ability and localization of the peptide, explored by flow cytometry and confocal laser scanning microscopy, respectively. The fluorophore, TAMRA, was chosen based on its net charge and high photostability. TAMRA is a zwitterionic compound with a neutral net charge at neutral pH which does not affect the overall net charge of P7 (Fischer et al., 2002). The unlabeled P7 behaves similarly to the labeled compound, as determined by the results of the MIC assay and flow cytometry. Both unlabeled P7 and TAMRA labeled P7 exhibited the antibacterial activity against *S.* Typhimurium with the same MIC. Treatment *S.* Typhimurium cells with 1 × MIC unlabeled P7 and TAMRA-labeled P7 for 30 and 60 min induced the same level of cell depolarization (Supplementary Figure 1). All peptides were purified by high-performance liquid chromatography (HPLC) and obtained as trifluoroacetate salts. The purity of all peptides was found to be greater than 98% as verified by analytical reverse-phase HPLC. The mass of these peptides were identified by electrospray ionization mass spectrometry (ESI-MS). TAMRA-labeled P7 had a molecular mass of 1992.52 Da with a mean hydrophobic moment (μH), of 0.801. All peptides including P7 and TAMRA-labeled P7 were resuspended in deionized water to obtain a stock concentration of 10 mg/ml and kept at −20°C.

### Bacterial Strains and Culture Conditions

*Salmonella enterica* serovar Typhimurium ATCC 13311, 24 drug-resistant foodborne and eleven drug-resistant human borne *S. enterica* serovar Typhimurium and 4,5,12:i:- isolates (Table 1) were used as test microorganisms. They were previously isolated for molecular characterization in 2014 and kindly provided by Dr. Soraya Chaturongakul (Mahidol University, Thailand) (Huoy et al., 2014). All bacteria were cultured in Tryptic Soy Broth (TSB) and incubated at 37°C for 18 h. Mueller Hinton Broth (MHB) was then used to wash and rinse the bacteria. The bacterial suspensions were then diluted to obtain concentrations of 10^7^ CFU/ml for inoculations.

**TABLE 1:** Minimum inhibitory concentration (MIC) and minimum bactericidal concentration (MBC) of P7 against antibiotic-sensitive and -resistant *Salmonella enterica* isolates.

**Strains**	**Serovar**	**Source**	**Drug susceptibility^*a*^**		**MIC (MBC) in μg/ml**	
			**Resistance**	**Intermediate**	**P7**	**Nisin**
**Standard strain**						
ATCC 13311	Typhimurium	ATCC	–	–	3.91 (3.91)	>1000
**Foodborne isolates**						
H2-009	4,5,12:i:-	Pork fat	Amp, C, S, T	–	3.91 (7.81)	>1000
H2-010	4,5,12:i:-	Pork skin	Amp, C, S, T	–	3.91 (7.81)	>1000
H2-038	4,5,12:i:-	Pork kidney	Amp, S, T	–	7.81 (7.81)	>1000
H2-039	4,5,12:i:-	Pork intestine	Amp, S, T	–	3.91 (3.91)	>1000
H2-042	4,5,12:i:-	Pork intestine	Amp, C, S, T, SxT	Cp	7.81 (7.81)	>1000
H2-043	4,5,12:i:-	Pork heart	Amp, S, T	Cp	7.81 (7.81)	>1000
H2-044	4,5,12:i:-	Pork kidney	Amp, C, S, T, SxT	Cp	7.81 (7.81)	>1000
H2-045	4,5,12:i:-	Pork intestine	Amp, S, T	Cp	7.81 (7.81)	>1000
H2-046	4,5,12:i:-	Pork heart	Amp, S, T	Cp	7.81 (31.25)	>1000
H2-047	4,5,12:i:-	Pork liver	Amp, S	Cp	7.81 (15.63)	>1000
H2-049	4,5,12:i:-	Pork intestine	Amp, S, T	–	3.91 (7.81)	>1000
H2-050	4,5,12:i:-	Pork heart	Amp, S	–	7.81 (15.63)	>1000
H2-054	4,5,12:i:-	Pork intestine	Amp, S, T	–	7.81 (7.81)	>1000
H2-055	4,5,12:i:-	Pork liver	Amp, S, T	–	7.81 (31.25)	>1000
H2-062	4,5,12:i:-	Pork intestine	Amp, S, T	Cp	7.81 (15.63)	>1000
H2-065	4,5,12:i:-	Pork liver	Amp, S, T	–	7.81 (31.25)	>1000
H2-067	4,5,12:i:-	Pork kidney	Amp, S, T	–	7.81 (15.63)	>1000
H2-070	4,5,12:i:-	Pork liver	Amp, Ctx, C, S, T, SxT	Cp	7.81 (7.81)	>1000
H2-071	4,5,12:i:-	Pork heart	Amp, Ctx, C, S, T, SxT	Cp	7.81 (15.63)	>1000
H2-072	4,5,12:i:-	Pork intestine	Amp, Ctx, C, S, T, SxT	Cp	7.81 (15.63)	>1000
H2-075	4,5,12:i:-	Pork intestine	Amp, S, T	–	7.81 (7.81)	>1000
H2-082	4,5,12:i:-	Pork intestine	Amp, S, T	–	7.81 (15.63)	>1000
H2-089	4,5,12:i:-	Pork intestine	Amp, S	–	7.81 (31.25)	>1000
H2-090	4,5,12:i:-	Pork intestine	Amp, S, T	–	7.81 (15.63)	>1000
**Clinical isolates**						
H1-001	Typhimurium	Blood	Amp, Ctx, C, Cp, NA, S, T	Amc	7.81 (15.63)	>1000
H1-006	Typhimurium	Blood	NA	S, Cp	7.81 (31.25)	>1000
H1-011	Typhimurium	Stool	Amp, S, T	Amc, Ctx, Cp, NA	7.81 (7.81)	>1000
H1-015	Typhimurium	Boot swab	Amp, S	Cp	7.81 (31.25)	>1000
H1-024	4,5,12:i:-	Rectal swab	Amc, Amp, Ctx, C, NA, S, SxT	Cp	3.91 (3.91)	>1000
H1-025	4,5,12:i:-	Urine	Amp, Ctx, C, S, T	Cp, NA, SxT	7.81 (31.25)	>1000
H1-027	4,5,12:i:-	Stool	Amp, S, T	–	7.81 (15.63)	>1000
H1-041	Typhimurium	Stool	Amp, Ctx, C, S, T, SxT	Amc, Cp, NA	7.81 (15.63)	>1000
H1-062	Typhimurium	Stool	Amc, Amp, Ctx, C, NA, S, T	Cp	7.81 (15.63)	>1000
H1-102	4,5,12:i:-	Stool	Amp, C, S, T, SxT	Amc	7.81 (7.81)	>1000
H1-100	4,5,12:i:-	Stool	Amp, C, Cp, S, T, SxT	Amc, NA	7.81 (7.81)	>1000

*Amc, amoxicillin/clavulinic acid; Amp, ampicillin; Ctx, cefotaxime; C, chloramphenicol; Cp, ciprofloxacin; NA, nalidixic acid; Nor, norfloxacin; S, streptomycin; T, tetracycline; SxT, sulfamethoxazole/trimethoprim.*

*^*a*^*In vitro* drug susceptibility of all isolates was performed using Kirby-Bauer disk diffusion test, in accordance with the standard procedure recommended by the Clinical and Laboratory Standards Institute (CLSI), and interpreted as susceptible, resistant, or intermediate according to interpretative criteria based on CLSI breakpoints (Clinical Laboratory Standards Institute [CLSI], 2018). The data were taken from Huoy et al. (2014).*

### Antibacterial Activity Assay

A modified version of the National Committee for Clinical Laboratory Standards (NCCLS) broth microdilution method (Steinberg et al., 1997) was used to determine the minimum inhibitory concentrations (MICs) of peptide and compared to that of nisin. Fifty microliters of two-fold serial dilutions of each peptide [0.98–250 μg/ml in 0.01% (v/v) acetic acid and 0.2% (w/v) bovine serum albumin (BSA, Sigma-Aldrich, MO, United States)], were incubated with 50 μl of bacterial suspensions (approximately 10^7^ CFU/ml) at 37°C for 24 h in a shaking incubator. MICs were taken as the lowest concentration of peptide at which growth was inhibited as observed by visual inspection. Bacterial cultures without peptides and uncultured broth were used as positive and negative controls, respectively. Minimal bactericidal concentrations (MBCs) were determined using a colony count assay (Clinical Laboratory Standards Institute [CLSI], 2015). Fifty microliters of samples from each non-turbid well in the MIC determination experiment was plated on Mueller Hinton Agar (MHA) and colonies were observed after overnight incubation at 37°C. MBCs were designated as the least concentration showing no colony growth on the plate. All of experiments were performed in triplicate.

### Time-Kill Kinetics Assay

The kinetics of P7 killing of bacteria was determined by observing the fraction of bacteria surviving peptide treatment during a period of time as described previously (Dong et al., 2018). Starting inoculations of *S. enterica* serovar Typhimurium (10^7^ CFU/ml) were incubated with P7 at 3.91 μg/ml (both MIC and MBC value are 3.91 μg/ml) in MHB at 37°C with continuous shaking at 200 rpm. At different time periods (0.5, 1, 2, 4, 6, and 24 h), the bacterial suspensions were 10-fold serially diluted in 1 × PBS, then 100 μl of each dilution was plated on MHA. Colonies of bacteria were counted after 24 h of incubation at 37°C and calculated as CFU/ml. The results were displayed as the mean of three independent experiments.

### Cytotoxicity Assay

The toxicity of P7 peptide on L929 mouse fibroblast cells was assessed by a dye reduction assay using 3-(4,5 dimethylthiazol-2-yl)-2,5-diphenyltetrazolium bromide (MTT). Briefly, 3 × 10^4^ L929 cells in RPMI 1640 [supplemented with 10% (v/v) fetal bovine serum, 0.2% (w/v) sodium bicarbonate, 2 mM L-glutamine, 100 U/ml penicillin and 100 mg/ml streptomycin] were seeded into each well of 96-well plates and then incubated in a humidified atmosphere with 5% CO_2_ at 37°C for 24 h. After the incubation period, the peptide was added to obtain final concentrations of 0.98–250 μg/ml; plates were incubated at 37°C under 5% CO_2_ for 24 h. RPMI 1640 medium with and without cells were used as positive and negative controls, respectively. After incubation, a final concentration of 0.5 mg/ml MTT (Sigma-Aldrich, MO, United States) was added and incubated at 37°C under 5% CO_2_ for 4 h. Then, the supernatants were removed from each well and 150 μl of dimethyl sulfoxide (DMSO) was added and mixed thoroughly to solubilize the formazan crystals. Subsequently, absorbance was measured at 570 nm using a microplate reader. The cell viability (%) was calculated according to the formula:

$$\%Cellviability=\frac{{\text{OD}}_{\text{Treated}}-O\,D_{B\,l\,a\,n\,k}}{{\text{OD}}_{\text{Untreated}}-O\,D_{B\,l\,a\,n\,k}}\times 100$$

### Circular Dichroism Spectroscopy

To investigate conformational changes of P7 induced by membrane-mimetic environments, secondary structure of the peptide was determined on a Jasco-815 spectropolarimeter (Jasco, Tokyo, Japan) in 0.1 cm pathlength rectangular quartz cell under nitrogen at 25°C (Liu et al., 2013). The peptide was prepared at a final concentration of 0.2 mg/ml in deionized (DI) water representing an aqueous surrounding, 30 mM SDS micelles to mimic negatively charged bacterial membranes and 50% (v/v) TFE (2,2,2-trifluoroethanol) to imitate hydrophobic part of bacterial membranes. Spectra were recorded in the 190–250 nm range at a scanning speed of 10 nm/min. At least three scans were recorded for each condition. Then, the acquired circular dichroism (CD) signal spectra were converted to mean residue ellipticity using the following equation:

θ=M(θ/o⁢b⁢s10)×(M/R⁢Wc⋅1)

where θ_*M*_ is residue ellipticity (deg. M^–1^ m^–1^), θ_*obs*_ is the measured ellipticity revised for the buffer at a given wavelength (mdeg), M_*RW*_ is average molecular weight of the residue (M_*W*_/number of amino acids), c is peptide concentration (mg/ml), and l is the path length (cm).

### Mode of Action of Peptide

#### Flow Cytometry Analysis

##### Membrane-penetrating activity

The penetration activity of P7 with bacterial cell membranes at different time interval was investigated by flow cytometry (Zhang et al., 2016). The mid-log bacterial suspensions (approximately 10^7^ CFU/ml in 1 × PBS) were incubated with 1 × MIC of TAMRA-labeled P7 at 37°C for 5, 10, 15, 30, 60, or 120 min. Subsequently, unbound labeled peptide was discarded by washing with 1 × PBS. The red fluorescence (585/42 nm) signal emitted by TAMRA fluorescence dye was evaluated by flow cytometry (CytoFlex, Beckman Coulter). 25,000 bacterial cells were counted in each sample. The experiments were done in triplicate and the data was analyzed by Kaluza software version 2.1 (Beckman Coulter, Brea, CA, United States).

##### Membrane permeability and depolarization

To further investigate the mechanism of membrane lytic activity, translational membrane potential dye or bis-(1,3-dibutylbarbituric acid) trimethine oxonol (BOX) and membrane impermeant propidium iodide (PI) were used to evaluate membrane depolarization and permeability induced by P7 as previously described (Rabanal et al., 2015). *S. enterica* serovar Typhimurium ATCC 13311 were diluted to 10^7^ CFU/ml and treated with P7 at concentrations of 1 × MIC, then incubated for 30, 60, and 120 min at 37°C with continuous shaking at 140 rpm. The treated bacterial cells were collected and washed with 1 × PBS. Afterward, PI (final concentration of 7.5 μg/ml, Sigma-Aldrich, MO, United States) and BOX (final concentration of 0.25 μM, Sigma-Aldrich, MO, United States) were added to each sample. Untreated bacterial cells (no peptide) and bacterial cells heated at 70°C for 30 min served as a negative and positive control, respectively. The fluorescent signal at a laser excitation wavelength of 488 nm was recorded using a flow cytometer (CytoFlex, Beckman Coulter). Forward scatter (FS), side scatter (SS), red fluorescence (585/42 nm) emitted by PI and green fluorescence (530/30 nm) from BOX were collected using logarithmic scales. In each sample, 25,000 bacterial cells defined in accordance with their scatter parameters, were counted. The experiments were carried out in triplicate and the data were analyzed using Kaluza software version 2.1 (Beckman Coulter, Brea, CA, United States).

#### Confocal Laser Scanning Microscopy (CLSM) Analysis

To investigate the localization of P7 on the bacterial membranes, confocal laser scanning microscopy was used to study *S. enterica* serovar Typhimurium ATCC 13311 after incubation with TAMRA-labeled P7 as described previously (Klubthawee et al., 2020). Bacteria cultures at mid-logarithmic phase were centrifuged at 2,000 × *g* for 5 min to remove the supernatants. Subsequently, the bacterial pellets were washed twice and resuspended in 1 × PBS. Bacterial inoculums (approximately 1 × 10^7^ CFU/ml) were incubated with 1 × MIC of TAMRA-labeled P7 at 37°C for 30 min. Afterward, unbound labeled peptide was discarded by rinsing with PBS. The samples were fixed with 4% paraformaldehyde for 20 min and washed twice in 1 × PBS. Samples were mixed with antifade (Prolong antifade reagent with DAPI; Invitrogen, CA, United States), follow by 2% low melting agarose (Sigma-Aldrich, MO, United States) on glass slides before observation under a confocal laser scanning microscopy (LSM 800 with Airyscan, ZEISS).

#### Scanning Electron Microscopy

The surface morphology of bacteria cells after treatment with P7 was observed under a scanning electron microscopy (SEM). As previously described (Lv et al., 2014), *S. enterica* serovar Typhimurium ATCC 13311 were cultured to logarithmic phase in TSB, washed twice and resuspended to an OD_620_ of 0.2 in 1 × PBS. The bacterial suspensions were incubated with 1 × MIC of P7 for 60 min at 37°C. After incubation, treated bacterial cells were centrifuged at 10,000 × *g* for 20 min and washed twice with 1 × PBS. Bacterial pellets were fixed in 2.5% (v/v) glutaraldehyde at 4°C overnight. After that, the samples were washed twice in PBS and dehydrated through a graded ethanol series (50, 70, 90, and 100%), for 15 min in each concentration. All the samples were then transferred to mixtures (1:1) of ethanol and tertiary butanol, and then to pure tertiary butanol, for 20 min each. After lyophilization and gold coating, the samples were examined using a scanning electron microscope (Hitachi SU8020, Tokyo, Japan).

#### Transmission Electron Microscopy (TEM)

Preparation of *S. enterica* serovar Typhimurium treated with P7 was performed in the same protocol as described for the SEM treatment. After fixing with 2.5% glutaraldehyde at 4°C overnight, the bacterial pellets were washed twice with PBS and post-fixed with 1% osmium tetroxide in PBS for 2 h. After washing the fixed bacterial cells twice with PBS, the dehydration for 15 min in a graded ethanol series (50, 70, 90, and 100%) was performed. After depositing in absolute acetone for 20 min, these samples were then transferred to a mixture of absolute acetone (1:1) and epoxy resin (1:3) for 1 h each, followed by transfer to pure epoxy resin and kept overnight. By cutting with a glass knife on an ultramicrotome, ultrathin sections were obtained and post-stained with uranyl acetate and lead citrate. Specimens were observed under a transmission electron microscope (Hitachi HT7700, Tokyo, Japan).

### Stability of Peptide

The stability of P7 was tested by determining its antibacterial activity after exposure to different environmental conditions (high salt concentration, different ranges of pH, proteolytic enzymes and high temperatures) as previously described (Shwaiki et al., 2020). To test the effect of salts on the antibacterial activity of P7, 10^7^ CFU/ml of *S. enterica* serovar Typhimurium ATCC 13311 was exposed to two-fold serially diluted concentrations (0.98–250 μg/ml) of P7 in the presence of different salts including NaCl (150 mM), KCl (4.5 mM), MgCl_2_ (1 mM), NH_4_Cl (6 μM), ZnCl_2_ (8 μM), FeCl_3_ (4 μM), and CaCl_2_ (2.5 μM) (Xu et al., 2014). To determine pH stability, the pH of the peptide’s solution was adjusted to 3, 5, 7, 9, and 11 using 1 M NaOH or 1 M acetic acid (CH_3_COOH). P7 were incubated in the different pH conditions for 1 h, then transferred to inoculated MHB for antibacterial testing (Chen et al., 2020), There was no change of the pH following the addition of peptide. To determine the sensitivity of peptide to proteolytic enzymes, the residual activity of P7 was measured after incubation the peptide with 1 mg/ml (final concentration) of proteolytic enzymes (pepsin or trypsin) at 37°C for 1 h and heat denaturation at 80°C for 1 h. To study thermostability, peptides were pre-incubated without bacteria at various temperatures (40, 60, 80, and 100°C) for 1 h and left to cool for 30 min before testing. After these treatments, the antibacterial assays were carried out and MICs determined as described in section “Antibacterial activity assay.”

### Effect of Heat on Membrane-Active Mechanism of Peptide

The effect of heat on the membrane-active mechanism of P7 was further investigated by flow cytometry. The peptide, P7, was pre-heated at different temperatures (40, 60, 80, and 100°C) for 1 h and left to cool for 30 min. Membrane permeability and depolarization activity were determined by incubating the pre-heated peptide (at MIC concentration) with *S. enterica* serovar Typhimurium ATCC 13311 cells and activity was determined as described in section “Membrane permeability and depolarization.”

### Experimental Induction of Bacterial Resistance to P7 Peptide

In order to induce bacterial resistance to P7, *S. enterica* serovar Typhimurium ATCC 13311 were cultured for 10 serial passages in MHB in the presence of P7 in a series of concentrations ranging from 0.98 to 250 μg/ml (Veldhuizen et al., 2013). In each passage, 50 μl of bacterial samples at sublethal concentrations (the highest peptide concentration displaying 80% growth compared with growth in MHB) were used to determine MIC values by a broth microdilution assay.

### Statistical Analysis

All experiments were performed in triplicate. The data were tested by one-way ANOVA and Tukey’s test with SPSS version 16.0. All results were presented as mean ± standard deviation (SD); *P*-value < 0.05 was considered statistically significant.

## Results

### Antibacterial Activity

The antibacterial activity of P7 compared to nisin was determined against *S. enterica* serovar Typhimurium ATCC 13311 and 35 drug-resistant *S. enterica* serovar Typhimurium and 4,5,12:i:- isolates in Thailand, including 24 foodborne and 11 clinical isolates (Table 1). P7 exhibited potent antibacterial activity to all tested strains including typical and drug-resistant isolates, with MICs and MBCs ranging from 3.91 to 7.81 μg/ml and 3.91 to 31.25 μg/ml, respectively. In contrast, nisin, an authorized food preservative, had no antibacterial activity against the tested microorganisms up to maximum concentration of 1 mg/ml.

### Bactericidal Kinetics

**FIGURE 1:**
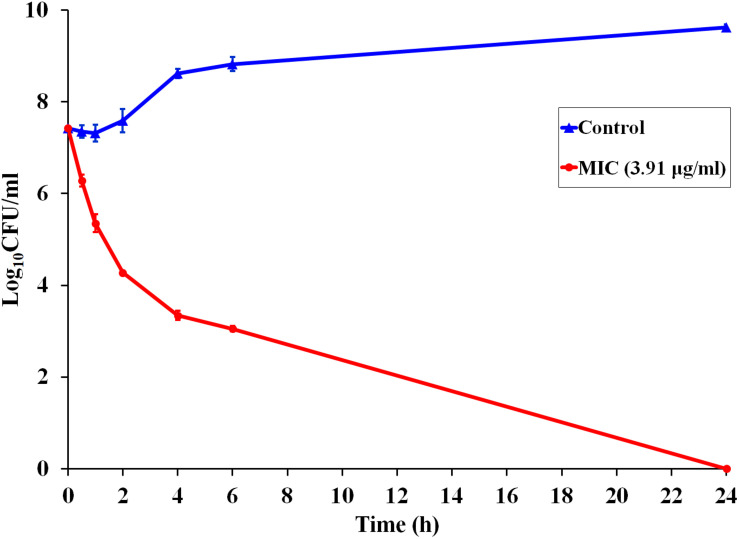
Time-dependent killing kinetics of P7 against *S. enterica* serovar Typhimurium ATCC 13311 at MIC concentration, exposed for 0.5, 1, 2, 4, 6, and 24 h.

Since P7 displayed robust antibacterial activity, a kinetic assay was performed to investigate the speed of the killing (Figure 1). At MIC concentration, P7 reduced the viable cells of *S. enterica* serovar Typhimurium more than 10^3^ and 10^4^CFU/ml within 2 and 4 h, respectively. After 24 h incubation, no bacteria were recovered. P7 exhibited rapid killing with time-dependent bactericidal action.

### Cytotoxicity

**FIGURE 2:**
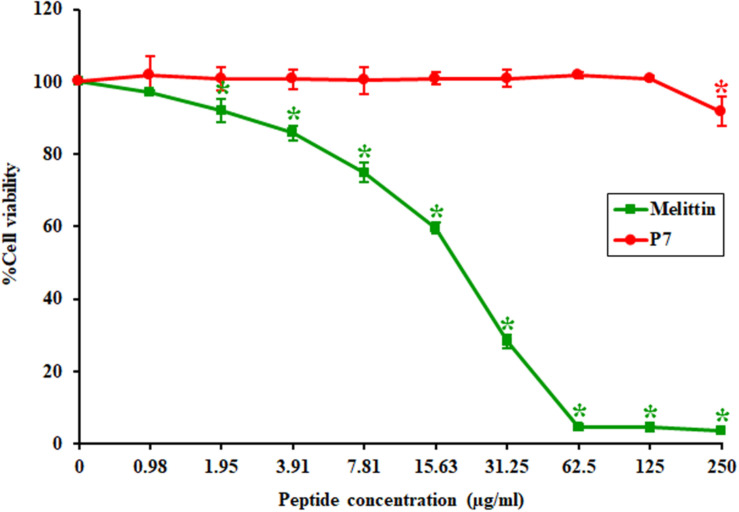
The toxicity of P7 and melittin against L929 mouse fibroblast cells. The results were obtained from three independent experiments; data were indicated as mean ± SD and the statistical analysis was carried out using one-way ANOVA and Tukey’s test (*p* < 0.05). Asterisk (*) indicates statistically significant difference (compared to untreated samples).

The toxicity of P7 to the L929 mouse fibroblast cell line was investigated using a colorimetric MTT viability assay. Melittin, a strong membrane-lytic peptide used as a positive control in the present study, showed strong cytotoxicity and dramatically reduced cell viability (to less than 10%) in the presence of 62.5 μg/ml (Figure 2). On the contrary, the P7 peptide had no cytotoxicity on L929 cells up to 125 μg/ml (Figure 2). Even at the maximum concentration tested (250 μg/ml), cell survival was up to 90%. This result was consistent with hemolytic activity reported previously (Klubthawee et al., 2020). Based on these findings together with the antibacterial results, P7 showed potent antimicrobial activity and a favorable safety profile.

### Secondary Structure Studies

**FIGURE 3:**
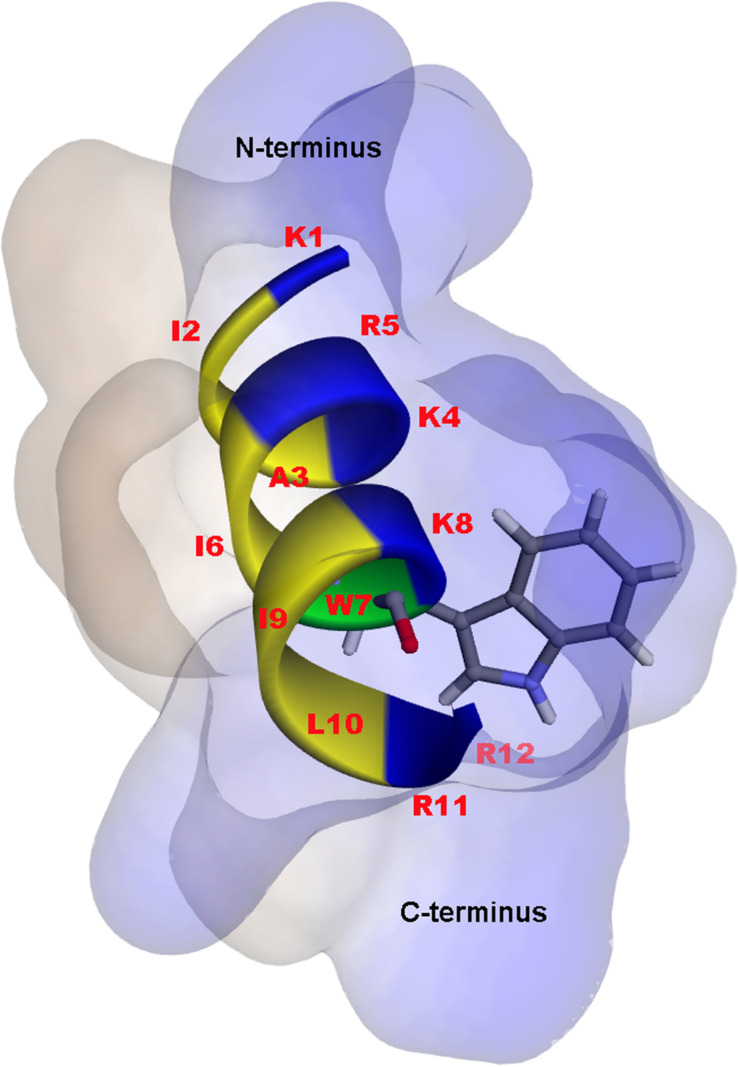
*In silico* three-dimensional (3D) molecular modeling, obtained by I-TASSER, with surface characteristics of the P7 structure from N- to C-terminus. Positively charged, hydrophobic and tryptophan residues are shown in blue, yellow, and green, respectively. Numbers represent the position of amino acid residues.

**FIGURE 4:**
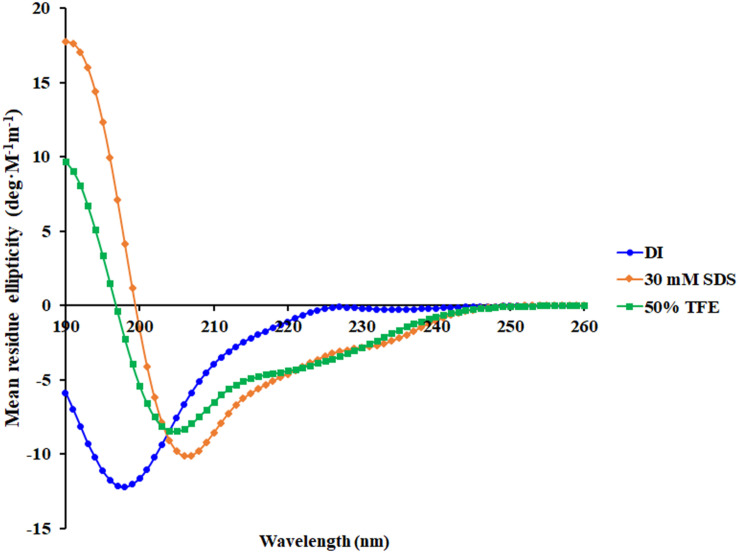
Conformational changes of P7 as determined by Circular dichroism (CD) spectroscopy using a Jasco-815 spectropolarimeter. CD spectra of P7 in aqueous solution, 30 mM SDS micelles, and 50% TFE are presented in blue, orange, and green, respectively.

A 3D structure of P7 was predicted by I-TASSER^1^ and an *in silico* 3D model was analyzed using BIOVIA Discovery Studio Visualizer (DSV) 2020. P7 formed an α-helical structure with a net positive charge of +6 and 50% hydrophobicity (Figure 3). As predicted by *in silico* molecular modeling, P7 displayed an amphiphilic α-helical conformation with a bulky side chain of tryptophan located on the hydrophilic face. CD spectroscopy was used to determine the conformation-activity relationships of the peptide in membrane-mimetic environments (Figure 4). Analysis of spectra acquired in a distilled water solution indicated that P7 displayed random coil or unordered conformation with a negative peak around 198 nm. In SDS micelles and 50% TFE environments, P7 demonstrated an α-helical secondary structure with a positive peak at approximately 190 nm and two negative peaks at approximately 208 and 222 nm. These results proposed that P7 formed an α-helical structure in membrane-mimetic environments.

### Membrane-Active Mechanism Determination

**FIGURE 5:**
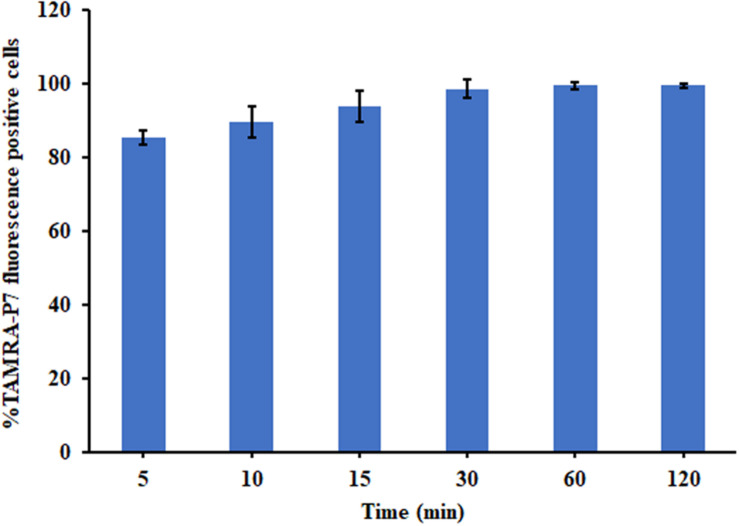
The membrane-penetrating action of TAMRA-labeled P7 to *S. enterica* serovar Typhimurium ATCC 13311 after 5, 10, 15, 30, 60, and 120 min of incubation as determined by flow cytometry. The experiments were done in triplicate and the data were represented as the mean ± SD.

**FIGURE 6:**
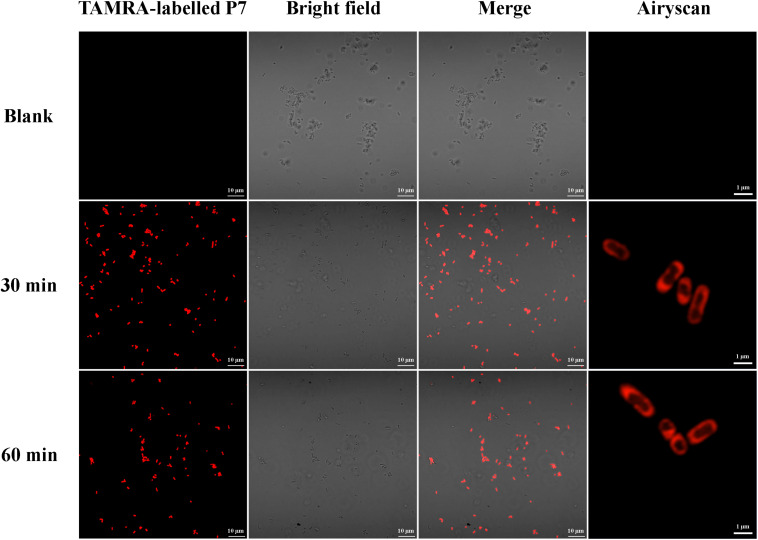
Localization of TAMRA-labeled P7 on *S. enterica* serovar Typhimurium ATCC 13311 at 30 and 60 min as observed by confocal microscopy.

The binding of P7 to bacterial membranes was determined by flow cytometry using TAMRA-labeled peptide. In the absence of TAMRA-labeled P7, no fluorescent signal was detected (Supplementary Figure 2) indicating no autofluorescence of the bacterial cells. After incubation, TAMRA-labeled P7-treated *S. enterica* serovar Typhimurium cells exhibited red fluorescence in a time-dependent manner (Figure 5). This was consistent with the results from confocal microscopy where TAMRA-labeled P7 showed an ability to penetrate *S. enterica* serovar Typhimurium cell membranes within 5 min (data not shown). It was observed that P7 peptides initially localized mainly in the membranes of *S. enterica* serovar Typhimurium, and subsequently penetrated and accumulated in the cytoplasm after 30 and 60 min of treatment (Figure 6). These results revealed that P7 exhibited rapid binding and penetration of *S. enterica* serovar Typhimurium in a time-dependent manner.

**FIGURE 7:**
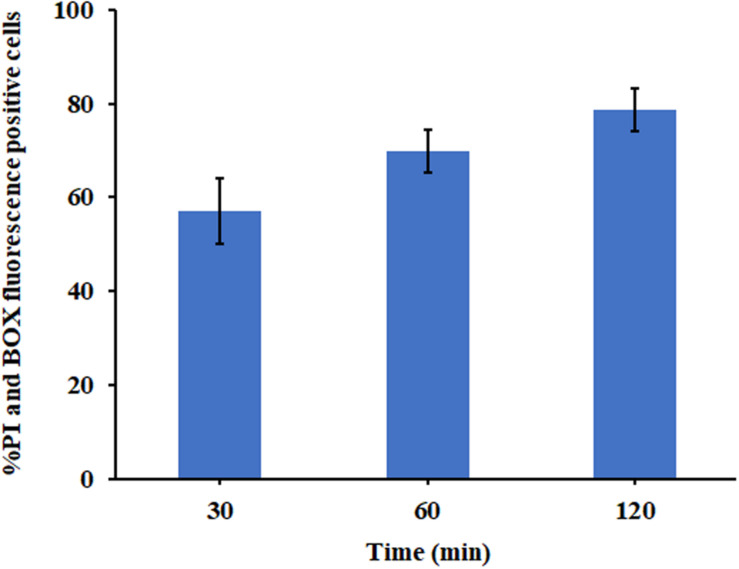
The effect of P7 on the membrane permeability (PI) and membrane potential (BOX) of *S. enterica* serovar Typhimurium ATCC 13311 as determined by flow cytometry. The experiments were performed in triplicate and the results were shown as the mean ± SD.

The ability of P7 to induce permeabilization of the bacterial membrane and depolarization of the cytoplasmic membrane of *S. enterica* serovar Typhimurium was assessed using DNA-staining PI, the fluorescence indicator of cell fatality based on the membrane permeability, and a voltage sensitive dye (BOX) which enters depolarized cells where it binds to lipid bilayers leading to the increase in fluorescence. Unstained *S. enterica* serovar Typhimurium were used to set the negative population. Without P7 treatment, most of *S. enterica* serovar Typhimurium bacteria showed neither PI nor BOX fluorescent signals, indicating that cell membranes were intact (Supplementary Figure 3). Heat at 70°C, a cooking temperature, was used as a positive control in the experiment as it is known to damage bacterial cell membranes (Ng et al., 2018). As expected, heat at 70°C induced high levels of permeabilization and depolarization at 1 × MIC for 30?min (Supplementary Figure 3). P7 at 1 × MIC treatment induced time-dependent permeabilization and depolarization of 57.10 ± 7.00%, 69.74 ± 4.54%, and 78.65 ± 4.49% of bacteria at 30, 60, and 120 min, respectively (Figure 7). These results suggested that P7 damaged the *S. enterica* serovar Typhimurium membranes via permeabilization and depolarization in time-dependent manner.

**FIGURE 8:**
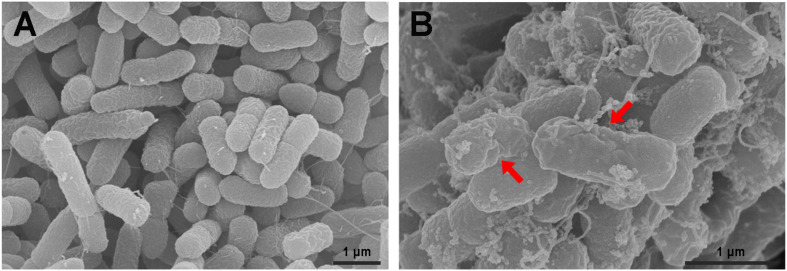
Scanning electron microscopic micrographs of *S. enterica* serovar Typhimurium ATCC 13311 treated with P7. Untreated bacterial cells **(A)** and treated with 1 × MIC of P7 for 60 min **(B)**. The red arrows indicate membrane roughening and corrugation.

**FIGURE 9:**
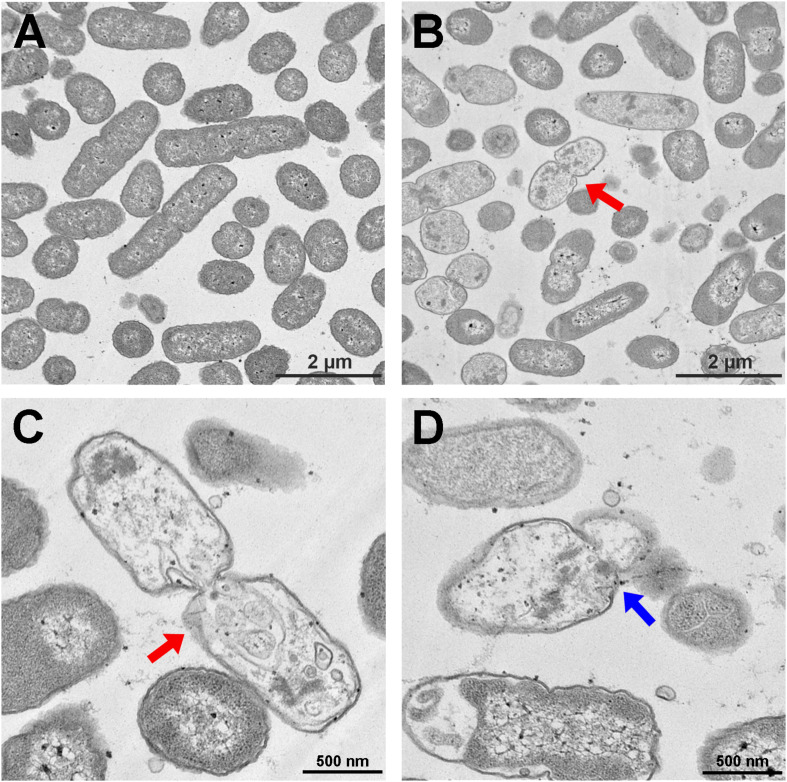
Transmission electron microscopic micrographs of *S. enterica* serovar Typhimurium ATCC 13311 treated with P7. Untreated bacterial cells **(A)** and treated with 1 × MIC of P7 for 60 min **(B–D)**. The red arrows indicate the disrupted cell membranes with an obvious cytoplasmic clear zone; blue arrow indicates visible pore and leakage of cellular contents.

The *S. enterica* serovar Typhimurium morphology and membrane integrity upon treatment with P7 were directly observed by SEM. These images demonstrated that the membrane surfaces of the *S. enterica* serovar Typhimurium cells treated with P7 displayed morphological changes, including significant membrane roughening and corrugation (Figure 8B) when compared with control *S. enterica* serovar Typhimurium cells which had bright, smooth surfaces or less surface roughness (Figure 8A). In parallel, TEM was performed to study membrane integrity and intracellular alterations of *S. enterica* serovar Typhimurium cells both before and after treatment with P7. As shown in Figure 9A, intact cell membranes and full intracellular contents were observed in untreated *S. enterica* serovar Typhimurium cells. In contrast, various structural alterations were observed after P7 treatment including obvious cytoplasmic clearance, disrupted cell membranes with detectable pores, and leakage of cellular compartments (Figures 9B–D).

### Peptide Stability

In general, various salts, proteolytic enzymes and pH all affect the *in vivo* antibacterial activity of AMPs. The MICs of P7 against *S. enterica* serovar Typhimurium ATCC 13311 in the presence and absence of different salts, pepsin and trypsin, and differing pH are shown in Tables 2, 3. In a salt-sensitive assay, P7 retained antimicrobial activity with a slight decrease in antimicrobial activity [two-fold increase of MICs (from 3.91 to 7.81 μg/ml)] in the presence of K^+^, NH_4_^+^, Zn^2+^, Fe^3+^, and Ca^2+^. The antimicrobial activity of P7 markedly decreased in the presence of Na^+^ and Mg^2+^ (about eight-fold increases in MICs and MBCs). These results suggested that P7 was more tolerant to K^+^, NH_4_^+^, Zn^2+^, Fe^3+^, and Ca^2+^ compared with Na^+^ and Mg^2+^. Therefore, some cations may compromise the antimicrobial activity of P7.

**TABLE 2:** MIC and MBC values (μg/ml) of P7 in the presence of various salts against *S. enterica* serovar Typhimurium ATCC 13311.

**Conditions**	**Salts^*a*^**							
	**Control^*b*^**	**NaCl**	**KCl**	**MgCl_2_**	**NH_4_Cl**	**ZnCl_2_**	**FeCl_3_**	**CaCl_2_**
MIC	3.91	31.25	7.81	31.25	7.81	7.81	7.81	7.81
MBC	3.91	125	31.25	125	31.25	62.5	31.25	31.25

*^*a*^The final concentrations tested were: NaCl (150 mM), KCl (4.5 mM), MgCl_2_ (1 mM), NH_4_Cl (6 μM), ZnCl_2_ (8 μM), FeCl_3_ (4 μM), and CaCl_2_ (2.5 μM).*

*^*b*^Control MIC values were determined in the absence of these salts.*

**TABLE 3:** MIC and MBC values (μg/ml) of P7 treated with different pHs, proteolytic enzymes and temperatures against *S. enterica* serovar Typhimurium ATCC 13311.

**Condition**	**pH**					**Proteolytic enzyme**		**Temperature (°C)**			
	**3**	**5**	**7**	**9**	**11**	**Pepsin**	**Trypsin**	**40**	**60**	**80**	**100**
***S. enterica* serovar Typhimurium ATCC 13311**											
MIC (μg/ml)	7.81	7.81	7.81	7.81	7.81	31.25	31.25	3.91	3.91	7.81	7.81
MBC (μg/ml)	15.63	7.81	7.81	7.81	15.63	31.25	31.25	3.91	7.81	7.81	7.81

Most AMPs are susceptible to protease degradation because their amino acid sequence serves as excellent substrates for proteolytic cleavage. As predicted by PeptideCutter^2^, the potential cleavage sites of P7 by pepsin and trypsin are the amino acids at positions 1, 4, 5, 8, 9, 11, and 12. The antibacterial activity of P7 after enzyme treatment was diminished. The MICs and MBCs against *S. enterica* serovar Typhimurium ATCC 13311 increased from 3.91 to 31.25 μg/ml indicating that the peptide was still active after proteolytic treatment.

A previous study demonstrated that pre-incubated PMAP-37(F34-R) in extreme pHs exhibited a stable and maintained antibacterial activity at pH 2-8 and pH 9-11 against *S. aureus* ATCC 25923 and *P. aeruginosa* GIM1.551 (Chen et al., 2020). Our results showed that P7 tolerated over a wide range of acidic, neutral even alkaline environment. After 1 h incubation in all tested pHs, ranging from pH 3 to pH 11 (Table 3), it exhibited potent antibacterial activity without significant change in MICs. Only two-fold increases in the MICs were observed across the pH range of 3–11.

### Effect of Heat on the Membrane-Active Mechanism of P7 Peptide

**FIGURE 10:**
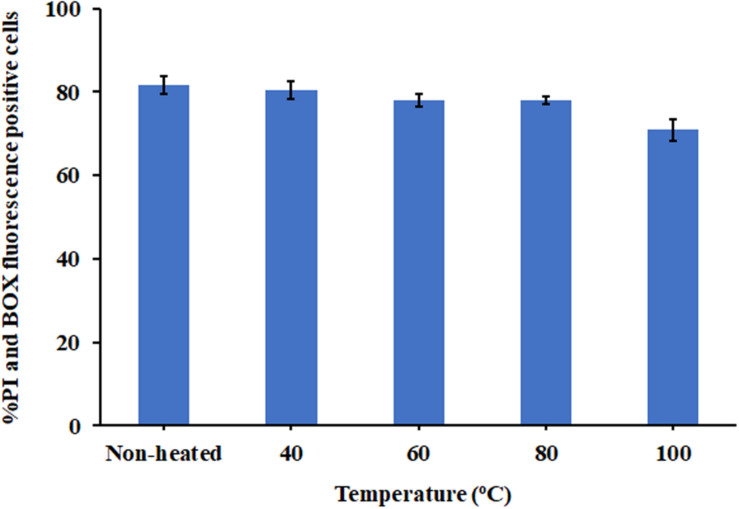
The effect of heat (40, 60, 80, and 100°C) on membrane-active mechanism of P7. Membrane permeability (PI) and depolarization activity (BOX) of pre-heated and non-heat peptide on *S. enterica* serovar Typhimurium ATCC 13311 were determined by flow cytometry. The results were obtained from three independent experiments and the data were presented as the mean ± SD.

Due to the intended use of P7 in food processing and preservation, it is important that it is able to withstand high temperatures, a common condition in food preparation. After being subjected to thermal treatment at 40 and 60°C for 1 h, no change in the MICs of the tested bacteria was observed (Table 3). Interestingly, the potent antibacterial activity was retained after 80 and 100°C treatment, with MICs and MBCs at 7.81 μg/ml. Results from flow cytometric analysis confirmed the membrane-active mechanism of P7 after temperature exposures of up to 100°C (Figure 10 and Supplementary Figure 4). P7, pre-heated at 40, 60, 80, and 100°C, potently induced membrane permeabilization and depolarization of the *S. enterica* serovar Typhimurium with 80.30 ± 2.13%, 77.91 ± 1.47%, 77.88 ± 0.88%, 70.84 ± 2.60%, respectively, which was comparable to that of non-heated peptide (81.69 ± 2.14%). These results demonstrated the thermostability of the peptide, increasing its potential for application in food processing and preservation.

### *In vitro* Induction of Peptide Resistance

The MICs were determined in 10 serial passages of bacteria treated with P7 (0.98–250 μg/ml). P7 retained killing activity through all bacterial passages with MIC of 3.91 μg/ml (Table 4). Thus, the potent antimicrobial activity of P7 was not compromised by induction of bacterial resistance *in vitro*.

**TABLE 4:** The MICs of P7 determined in 10 serial passages of bacterial cultures.

**Bacterial strain**	**MIC (μg/ml)**									
	**Serial passages^*a*^**									
	**1**	**2**	**3**	**4**	**5**	**6**	**7**	**8**	**9**	**10**
*S. enterica* serovar Typhimurium ATCC 13311	3.91	3.91	3.91	3.91	3.91	3.91	3.91	3.91	3.91	3.91

*^*a*^MIC values were determined in serial passages of bacterial cultures treated with P7. Bacteria were grown overnight in the presence of 0.98–250 μg/ml peptide. The sample containing the highest P7 concentration showing 80% bacterial growth was subsequently sub-cultured into new medium containing 0.98–250 μg/ml P7 peptide for determining MIC values by a broth microdilution assay. This procedure was repeated for 10 serial passages cultures.*

## Discussion

We have previously shown that P7, a 12-mer cationic peptide, modified from the cathelicidin family, displays broad-spectrum antibacterial activity against both Gram-positive and Gram-negative bacteria, and no toxicity against hRBCs (Klubthawee et al., 2020). In this study, P7 displayed a rapid bacterial-killing ability which was time-dependent and had a low bactericidal concentration against *S. enterica* serovar Typhimurium ATCC 13311. It was effective against the traditionally antibiotic-resistant *S. enterica* serovar Typhimurium and its monophasic variant strains isolated from food and humans. Though it had potent antimicrobial activity, P7 showed no obvious toxicity against L929 mouse fibroblast cells even at the maximum concentration tested. The positive charge of AMPs causes them to bind preferably to the negatively charged bacterial membranes which contain acidic phospholipids and anionic molecules, such as phosphatidylglycerol, cardiolipin, and lipopolysaccharide (Matsuzaki, 1999; Epand et al., 2006). In the case of mammalian cells, acidic phospholipids are usually sequestered in the inner leaflets of plasma membranes, while the outer leaflets are largely consisted of zwitterionic phosphatidylcholine and sphingomyelin (Matsuzaki, 2009).

Consistent with the predicted results from *in silico* three-dimensional structure models, P7 was converted from a random coil in aqueous environments to an α-helical structure in membrane-mimetic environments (50% TFE environments mimicking the hydrophobic environment of a microbial membrane and 30 mM SDS environments comparable to the negatively charged prokaryotic membrane) as shown by CD analysis. This implied that the negatively charged bacterial membrane and their hydrophobicity part is the key to antimicrobial activity of the α-helical cationic P7 peptides. As previously described (Brogden, 2005), the anionic components on bacterial outer surface and membranes play a crucial role in inducing the conformational change of peptide which is necessary for peptide attachment and insertion into the hydrophobic part of membrane.

Although the exact mechanism of action of AMPs remains controversial, most AMPs kill bacteria by disrupting the cell membrane (Lorenzon et al., 2019). As previously described, the first steps of AMP-mediated killing are attraction and attachment which initially occur via electrostatic interactions between the cationic peptides and the negatively charged surfaces of bacteria, after which they interact with the outer membrane (Brogden, 2005). To evaluate this possibility, we first used flow cytometry to assess the ability of P7 to penetrate the membrane of *S. enterica* serovar Typhimurium employing TAMRA-labeled P7. The binding activity of P7 was studied using TAMRA-labeled P7; the target site was visualized by confocal microscopy. The results revealed that P7 exhibited rapid binding activity, membrane penetration and then accumulation in *S. enterica* serovar Typhimurium cytoplasm in a time-dependent manner. Once peptides insert into the lipid bilayers, they can permeabilize and depolarize bacterial membranes. The results were indistinguishable from those of the positive control group (heating at 70°C) in which high temperature had damaged the bacteria by denaturizing the membrane proteins resulting in the release of intracellular organelles and cell lysis (Ebrahimi et al., 2018). After attachment, P7 at threshold concentration induced membrane permeabilization and depolarization in a time-dependent manner as observed by flow cytometry. SEM and TEM results confirmed that P7 caused damage to the *S. enterica* serovar Typhimurium membranes via extensive membrane rupture and pore formation, resulting in the leakage of intracellular contents and cell death. In accordance with previous reports (Sani and Separovic, 2016; Dong et al., 2018), these results illustrate that the initial step in the antimicrobial activity of P7 was the ability to selectively bind the anionic lipids of the bacterial outer membrane and subsequently insert into the hydrophobic core of the membrane bilayer, ultimately disrupting the bilayer’s integrity.

The initial electrostatic interaction of positively charged AMPs with the negatively charged surface of bacteria is a key of antimicrobial activity. Free ions or other positively charged molecules, including salts, can weaken this electrostatic interaction and decrease the efficiency of this antimicrobial activity (Ma et al., 2016). P7 maintained its inhibitory activity in the presence of K^+^, NH_4_^+^, Zn^2+^, Fe^3+^, and Ca^2+^. This might be due to the bulky side chain of tryptophan, located on the peptide’s hydrophilic face and containing positively charged arginine and lysine, enhancing the binding, attachment and insertion of the P7 peptide into the bacterial membrane and so maintaining its antibacterial activity in the presence of salts (Lawyer et al., 1996; Saravanan et al., 2014). Nonetheless, the effects of cations likely rely on both the particular peptide and the concentration of salt (Ma et al., 2015). At higher millimolar concentrations, Na^+^ (150 mM) and Mg^2+^ (1 mM) caused almost a 10-fold reduction in the MIC of P7, while most of the other salts (micromolar range) caused only a twofold reduction in the MIC. The inhibitory effects of monovalent Na^+^ may rely solely on interruption of the electrostatic attraction, resulting in decreased binding efficacy of peptide to negatively charged membranes (Huang et al., 2011). Mg^2+^ may weaken the inhibitory activity of P7 not only by interfering with the electrostatic attraction, but also by initiating membrane binding competition between peptides and cations on bacterial outer membranes (Slochower et al., 2014; Ma et al., 2015). Chou et al. (2019) reported that F4 peptide retained killing activity in the presence of salts at physiological concentrations except for Na^+^ and Mg^2+^, while still displaying effective antimicrobial potency in the mouse model. Notably, P7 lost part of its killing activity at physiological salt concentrations as shown by MBC values which were 10–40x higher than those of control. There are some cationic AMPs which show partial or even complete loss of antimicrobial activity at physiological salt concentrations (Scudiero et al., 2010; Mai et al., 2011).

In general, microorganisms are sensitive to a food’s pH, and very low or very high pH values will prevent the growth of microorganisms. However, very few foods have pH values low or high enough to completely inhibit microbial growth or offer much preservative value (McGlynn et al., 2003). For almost all foods, a combination of preservative agents and food processing must be used to help preserve the food. Here, our aim was to identify new preservative agents with ideal characteristics such as the ability to endure extreme conditions (heat and wide pH range). The P7 peptide is stable over a wide range of pHs (pH 3–11) and still maintained its antibacterial activity after exposure to an acidic or basic environment, underlining its potential application in food preservation. During food processing, high temperatures might denature or change the specific configuration of a peptide which is necessary for its antibacterial activity (Jabeen and Khanum, 2017). To determine whether the peptide bonds in P7 were broken at high temperature and resulted in reduced activity, the peptides were pre-incubated without bacteria at various temperatures. The results revealed that P7 maintained its potent antibacterial activity (via induced membrane permeabilization and depolarization) even after treatment at 100°C for 1 h. These results highlighted the high thermostability of P7 peptide, important for its potential use in the food industry.

In the use of peptides as food preservatives, their degradation products after digestion should be considered. Catabolism of AMPs is governed by the sequence of their amino acids and these products can serve as nutrients after digestion (Chakrabarti et al., 2018). During the digestion process, pepsin (the primary proteolytic enzyme in the stomach) and trypsin (produced in the pancreas and secreted into the small intestine) act on ingested peptides and break them into smaller peptides which can be readily absorbed across the intestine’s epithelial barrier (Patel and Misra, 2011). Although P7 peptide showed strong heat stability, which is beneficial during food processing, they were shown to be sensitive to proteolytic digestion. The MICs and MBCs of P7 against *S. enterica* serovar Typhimurium ATCC 13311 increased approximately eight-fold in the experimental *in vitro* pepsin and trypsin enzyme conditions. Therefore, the degradation of this peptide by proteolytic enzymes of the intestinal tract, when ingested as a food preservative, would restrict its antibacterial activity against gastrointestinal microbiota and decrease potential adverse effects on health (Thery et al., 2019).

Besides potent antimicrobial activity and good thermal stability, the tendency of the peptide to induce bacterial resistance was necessarily a concern. It is widely speculated that bacterial resistance will be acquired when they are exposed to long-term sublethal concentrations of antimicrobials. Thus, the MICs of P7 against 10 serial passages of bacteria were determined in this study. Peptide P7 retained its killing activity at 3.91 μg/ml through all bacterial passages. These results suggested that the ability of *S. enterica* serovar Typhimurium to develop resistance to P7 was low, similar to the chicken cathelicidins (Veldhuizen et al., 2013). This might result from the rapid bactericidal activity of P7, which induced membrane damage and cell lysis, and thus make it difficult for bacteria to develop resistance (Park et al., 2011). Taken together, these findings highlighted the potent antimicrobial activity of P7 without its inducing bacterial resistance and its potential as an alternative food preservative.

## Conclusion

This study highlighted on the potential use of the highly thermostable membrane-permeabilizing peptide, P7, especially against drug-resistant foodborne *S. enterica* serovar Typhimurium and its monophasic variant spreading within the food industry. P7 kills bacteria via membrane attraction and attachment followed by infiltration through the bacteria’s membrane, inducing membrane depolarization and permeabilization, membrane disruption and subsequent leakage of intracellular composition and cell death. Moreover, this peptide appears unlikely to induce bacterial resistance. Although this study reflected the situation at an early *in vitro* stage, the properties, characteristics and mode of action of peptide described here will be benefit for future developments and *in vivo* modeling. The work advances the ultimate aim of exploiting the peptide’s antibacterial potential for improved food processing and preservation.

## Data Availability Statement

The original contributions presented in the study are included in the article/Supplementary Material, further inquiries can be directed to the corresponding author/s.

## Author Contributions

NK: investigation, methodology, and writing – original draft. RA: conceptualization, funding acquisition, investigation, validation, writing – original draft, and writing – review and editing. Both authors contributed to the article and approved the submitted version.

## Conflict of Interest

The authors declare that the research was conducted in the absence of any commercial or financial relationships that could be construed as a potential conflict of interest.

## Footnotes

1 http://zhanglab.ccmb.med.umich.edu/I-TASSER/

2 https://web.expasy.org/peptide_cutter/

## Supplementary Material

Supplementary Figure 1 Flow cytometry analysis of *S. enterica* serovar Typhimurium ATCC 13311 treated with TAMRA-labeled P7. *S. enterica* serovar Typhimurium cell population **(A)**. Untreated bacterial cells with TAMRA dye **(B)** and BOX staining **(C)**. The effect of TAMRA-labeled P7 at 1 × MIC for 30 and 60 min **(D,E)** on the membrane potential (BOX) of *S. enterica* serovar Typhimurium.

Supplementary Figure 2 Binding of TAMRA-labeled P7 at 1 × MIC to *S. enterica* serovar Typhimurium ATCC 13311 as measured by flow cytometry. *S. enterica* serovar Typhimurium cell population **(A)**. Untreated bacterial cells without staining **(B)**. Untreated bacterial cells stained with TAMRA fluorescence dye **(C)**. The membrane-penetrating activity of TAMRA-labeled P7 after 5 min **(D)**, 10 min **(E)**, 15 min **(F)**, 30 min **(G)**, 60 min **(H),** and 120 min **(I)** incubation. The numbers in the center of each plot represented the percentage of cell populations.

Supplementary Figure 3 Flow cytometry analysis of *S. enterica* serovar Typhimurium ATCC 13311 treated with P7. Untreated bacterial cells without PI and BOX **(A)**. Untreated bacterial cells with PI and BOX staining **(B)**. The effect of thermal lysis at 70°C for 30 min (positive control) **(C)**, and P7 at 1 × MIC for 30, 60, and 120 min **(D–F)** on the membrane permeability (PI) and membrane potential (BOX) of *S. enterica* serovar Typhimurium.

Supplementary Figure 4 Flow cytometry analysis of *S. enterica* serovar Typhimurium ATCC 13311 treated with preheated P7 at various temperatures for 2 h before testing. *S. enterica* serovar Typhimurium cell population **(A)**. Untreated bacterial cells without PI and BOX staining **(B)**. Untreated bacterial cells with PI and BOX staining **(C)**. Bacterial cells treated with P7 at 1 × MIC as control **(D)**. The effect of heat (40, 60, 80, and 100°C) to P7 on its membrane-active mechanism (**E,F,G,H**, respectively).
