# Supplementary material for: A Thermostable, Modified Cathelicidin-Derived Peptide With Enhanced Membrane-Active Activity Against Salmonella enterica serovar Typhimurium
Source: Front Microbiol. 2021 Jan 13;11:592220. doi: 10.3389/fmicb.2020.592220 (PMC7838546; doi:10.3389/fmicb.2020.592220)
Supplement: Supplementary Figure 1 — Flow cytometry analysis of S. enterica serovar Typhimurium ATCC 13311 treated with TAMRA-labeled P7. S. enterica serovar Typhimurium cell population (A). Untreated bacterial cells with TAMRA dye (B) and BOX staining (C). The effect of TAMRA-labeled P7 at 1 × MIC for 30 and 60 min (D,E) on the membrane potential (BOX) of S. enterica serovar Typhimurium. [file Data_Sheet_1.docx]

Supplementary Material


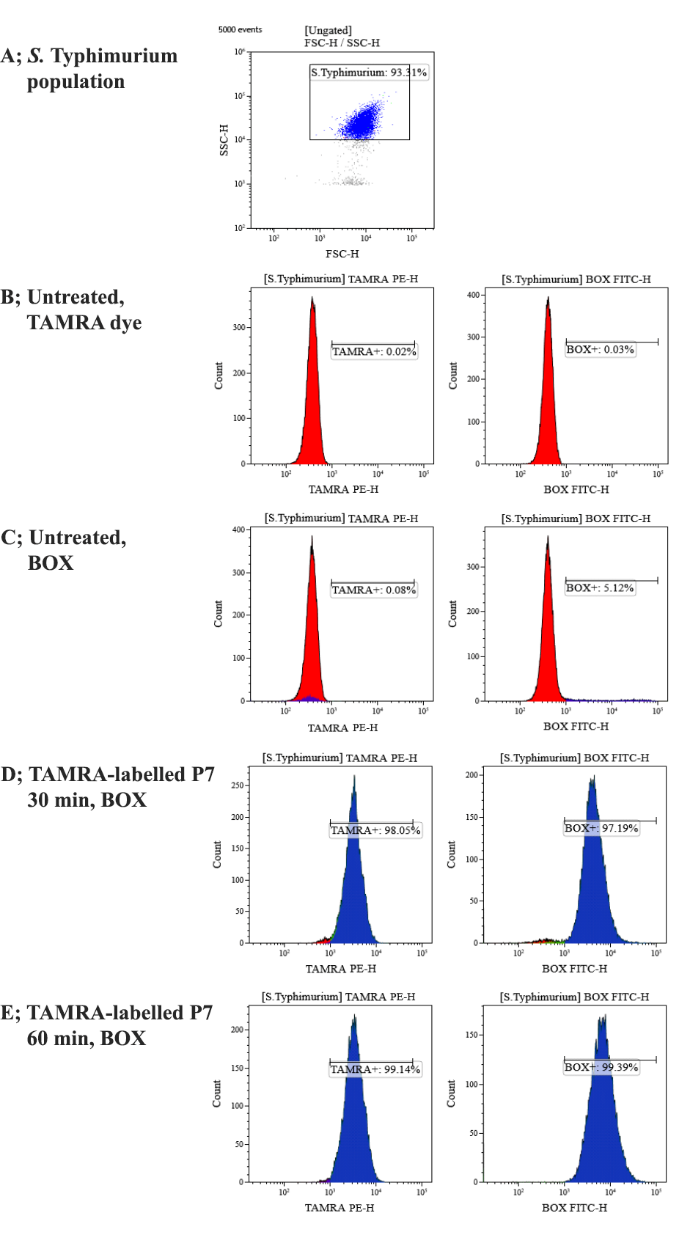


**Supplementary Figure 1.** Flow cytometry analysis of *S. enterica* serovar Typhimurium ATCC 13311 treated with TAMRA-labelled P7. *S.* *enterica* serovar Typhimurium cell population (A). Untreated bacterial cells with TAMRA dye (B) and BOX staining (C). The effect of TAMRA-labelled P7 at 1 × MIC for 30 and 60 min (D and E) on the membrane potential (BOX) of *S. enterica* serovar Typhimurium.


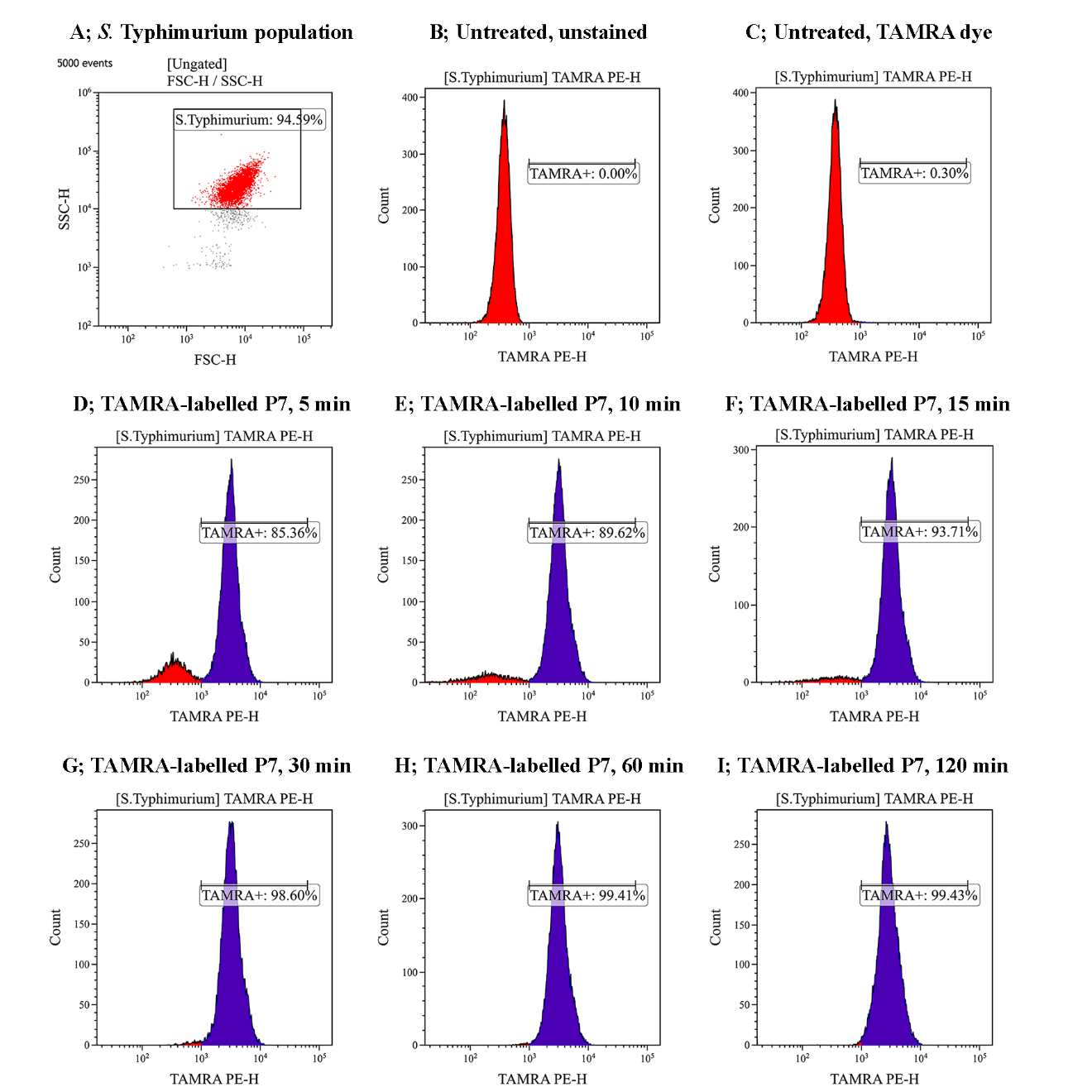


**Supplementary Figure 2.** Binding of TAMRA-labelled P7 at 1 × MIC to *S. enterica* serovar Typhimurium ATCC 13311 as measured by flow cytometry. *S. enterica* serovar Typhimurium cell population (A). Untreated bacterial cells without staining (B). Untreated bacterial cells stained with TAMRA fluorescence dye (C). The membrane-penetrating activity of TAMRA-labelled P7 after 5 min (D), 10 min (E), 15 min (F), 30 min (G), 60 min (H) and 120 min (I) incubation. The numbers in the center of each plot represented the percentage of cell populations.


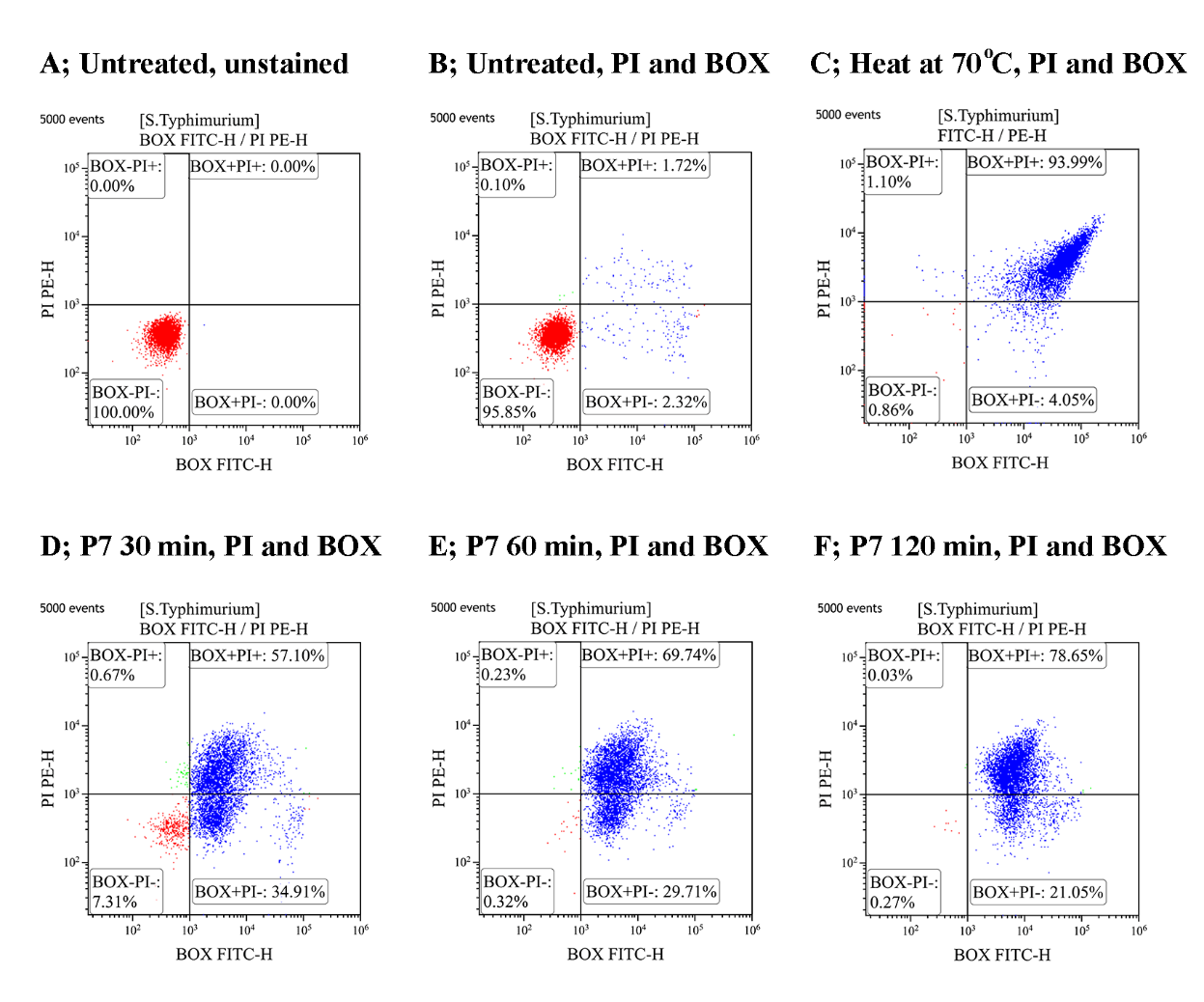


**Supplementary Figure 3.** Flow cytometry analysis of *S. enterica* serovar Typhimurium ATCC 13311 treated with P7. Untreated bacterial cells without PI and BOX (A). Untreated bacterial cells with PI and BOX staining (B). The effect of thermal lysis at 70°C for 30 min (positive control) (C), and P7 at 1 × MIC for 30, 60 and 120 min (D-F) on the membrane permeability (PI) and membrane potential (BOX) of *S. enterica* serovar Typhimurium.


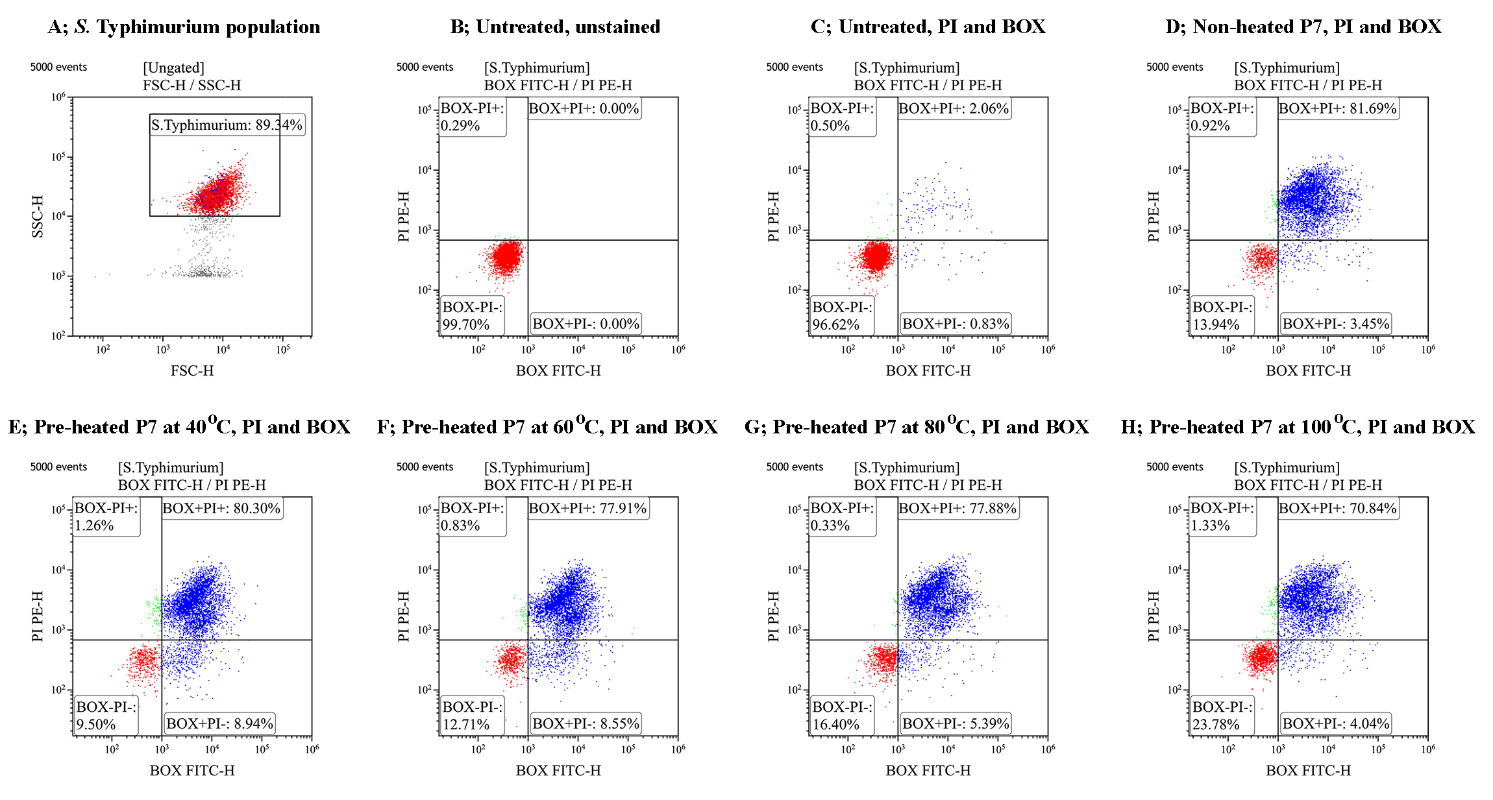


**Supplementary Figure 4.** Flow cytometry analysis of *S. enterica* serovar Typhimurium ATCC 13311 treated with preheated P7 at various temperatures for 2 h before testing. *S. enterica* serovar Typhimurium cell population (A). Untreated bacterial cells without PI and BOX staining (B). Untreated bacterial cells with PI and BOX staining (C). Bacterial cells treated with P7 at 1 × MIC as control (D). The effect of heat (40°C, 60°C, 80°C and 100°C) to P7 on its membrane-active mechanism (E-H, respectively).
